# Cerebrotendinous xanthomatosis‐associated diarrhea and response to chenodeoxycholic acid treatment

**DOI:** 10.1002/jmd2.12163

**Published:** 2020-08-30

**Authors:** Eric P. Brass, Bianca M.L. Stelten, Aad Verrips

**Affiliations:** ^1^ Department of Medicine, David Geffen School of Medicine at UCLA Los Angeles California USA; ^2^ Department of Neurology Catharina Hospital Eindhoven The Netherlands; ^3^ Department of Neurology Canisius Wilhelmina Hospital Nijmegen The Netherlands

**Keywords:** Cerebrotendinous xanthomatosis (CTX), Chenodeoxycholic acid (CDCA), diarrhea

## Abstract

**Background:**

In patients with cerebrotendinous xanthomatosis (CTX), chronic diarrhea is one of the earliest and main symptoms of the disease. In the current study, we evaluated the characteristics of the diarrhea and its response to chenodeoxycholic acid (CDCA) therapy in a cohort of Dutch CTX patients.

**Methods:**

We performed a retrospective review of medical records for 33 genetically confirmed CTX patients, and abstracted the characteristics of the diarrhea and the response to CDCA therapy (15 mg/kg/day up to 750 mg/day). The Bristol Stool Scale (BSS) was used for qualitative characterization of the stool.

**Results:**

Twenty‐five patients had diarrhea documented at baseline (76%). Of these patients, 10 had diarrhea rated as 6 (fluffy pieces with ragged edges, a mushy stool), and 6 had diarrhea rated as 7 (watery, no solid pieces, entirely liquid) using the BSS. In 10 patients for whom data were recorded, the median stool frequency at baseline was 3 per day (range 2‐6 per day). The response rate with CDCA for diarrhea resolution was 100% based on at least one post‐baseline visit without diarrhea and 95% as assessed at the first post‐baseline visit. In 68% of cases resolution was complete and sustained as no episodes of diarrhea were documented for follow‐up periods as long as 25 years.

**Conclusions:**

Chronic diarrhea persisting for years without spontaneous remission is a common feature of CTX at diagnosis. Chenodeoxycholic acid is an effective treatment for symptomatic relief of diarrhea in patients with CTX.

## 1 INTRODUCTION

Cerebrotendinous xanthomatosis (CTX; OMIM #213700) is an autosomal recessive disorder due to mutations of the *CYP27A1* gene, resulting in a deficiency of sterol‐27‐hydroxylase.^1^ The clinical presentation of CTX is heterogeneous and ranges from being nearly asymptomatic in early childhood up to severe disability secondary to progressive neurological dysfunction at adult age. Early diagnosis and initiation of treatment with chenodeoxycholic acid (CDCA) is felt important in preventing the progression of neurologic deterioration.^2^, ^3^ Chronic diarrhea is one of the earliest symptoms in CTX, typically beginning in childhood, and may be the only symptom present for many years in these patients.^2^, ^4^, ^5^, ^6^, ^7^, ^8^, ^9^, ^10^, ^11^ However, relatively limited information is available on its natural history or granular features. In the current study, we evaluated the characteristics of diarrhea and its response to CDCA, in a cohort of Dutch CTX patients.

## 2 METHODS

### 2.1 Data abstraction

Medical records of 33 Dutch patients presenting to the Neurology Clinic at Canisius‐Wilhelmina Hospital from 1981 until 2014, diagnosed with CTX and for whom pre‐treatment data were available, were reviewed. The Neurology Clinic is a national referral center for the care of patients with CTX, and pre‐diagnosis medical records were available for a subset of the cohort. All patients were evaluated at visits by one of the authors (AV) in a systematic manner and data recorded in the medical chart. The patients reported here were included in previous publications focussing on other clinical aspects of CTX.^2^, ^12^

The records for all visits for each patient were reviewed to determine if diarrhea was assessed. If a specific entry documented the presence of diarrhea, the patient was included as having diarrhea at that visit. If an entry documented the absence of diarrhea the patient was included as not having diarrhea at the visit. If no specific entry addressed diarrhea than the visit did not contribute to the analysis. Additionally, if the record contained information on stool quality, this was scored using the Bristol Stool Scale (BSS).^13^ Similarly, if the number of stools per day was available, these data were abstracted.

### 2.2 Patient diagnosis and management

The diagnosis of CTX was based on history, family history, clinical examination, and confirmed by measurement of plasma cholestanol concentrations and/or urine bile alcohol excretion, and genotyping in all patients.

Treatment with CDCA was initiated in all patients. The starting dose was 15 mg/kg/day to a maximum of 750 mg/day, divided into three equal doses per day. The dose was adjusted based on tolerability and response to the therapy as assessed by plasma cholestanol concentrations, urine bile alcohol excretion, and clinical findings. Follow‐up visits at the Neurology Clinic were done on an as‐needed basis without a fixed timing.

The clinical visit at which CDCA treatment was initiated was considered baseline. Antecedent visits were considered as pre‐baseline, and subsequent visits were considered as post‐baseline. If plasma cholestanol was not measured at the baseline visit the value from the visit closest to baseline was used as the baseline assessment.

### 2.3 Data presentation

Data for continuous variables are presented as mean ± SD, and categorical variables as percentages. Medians and ranges are used for datasets with skewed distributions. Where data elements were unavailable in the medical record no attempt was made to impute values. No prospective hypotheses were developed, and thus no P‐values are reported.

## 3 RESULTS

### 3.1 Cohort characteristics at baseline

Baseline characteristics are shown in Table 1. The mean age at baseline was 27 years. Twenty‐five of the 33 patients (76%) had diarrhea documented at baseline. Patients with diarrhea averaged 25 ± 13 years of age (N = 25) as compared with 33 ± 16 years of age (N = 8) for those without diarrhea. Of note, the cohort without diarrhea included an infant screened based on family history and which skewed the cohort's means for age. Excluding this infant, patients without diarrhea were 37 ± 10 years of age (N = 7).

**TABLE 1:** Baseline characteristics of 33 genetically confirmed Dutch CTX patients

	Age at baseline (years)	Sex (M/F)	Bristol Stool Scale	Stools per day	Cholestanol (μmol/L)^a^
All patients (Includes patients with and without diarrhea)	27 ± 14 (N = 33)	20/13	N/A	N/A	81 ± 57 (N = 28)
Patients without diarrhea	33 ± 16 (N = 8)	4/4	N/A	N/A	71 ± 37 (N = 6)
Patients with diarrhea	25 ± 13 (N = 25)	16/9	Score 7 (N = 6) Score 6 (N = 10)	3 [2–6] (N = 10)	84 ± 62 (N = 22)

*Note:* Values are mean ± SD or medians with range in brackets.

Abbreviations: N, number of patients, shown in parentheses; N/A, not applicable.

^a^ Upper limit of normal for plasma cholestanol is 12.5 μmol/L.

Sixteen medical records included a description of the stool at baseline which permitted assignment of a score using the BSS.^13^ Ten of these cases were rated as 6 (fluffy pieces with ragged edges, a mushy stool) and six were rated as 7 (watery, no solid pieces, entirely liquid). In 10 records the stool frequency was noted with a median of 3 stools per day (range 2‐6 per day).

**FIGURE 1:**
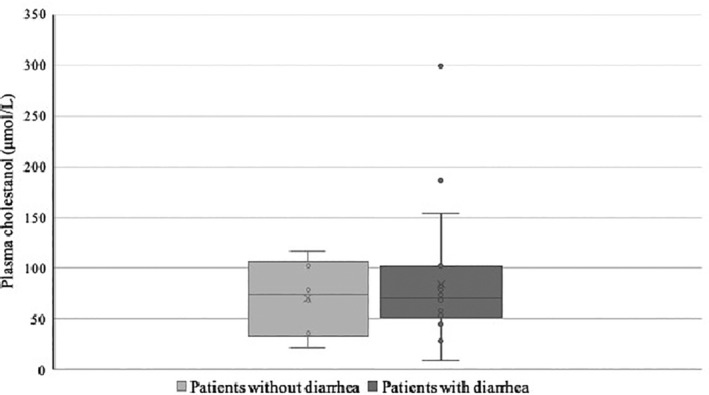
Plasma cholestanol concentration (μmol/L) at baseline. Box and whisker plot with the median value shown as the horizontal line, the box shows the medians for the range of the first and third quartiles, the whiskers the range except for outliers which are shown as individual points. Patients were separated into those with (N = 22) and without (N = 6) diarrhea at baseline. Upper limit of normal for the cholestanol assay is 12.5 μmol/L

Patients with diarrhea had mean plasma cholestanol concentrations at baseline of 84 ± 62 μmol/L with a median of 69 μmol/L (n = 22). This compares with a mean of 71 ± 37 μmol/L and a median of 79 μmol/L (n = 6) in patients without diarrhea (Table 1, Figure 1).

### 3.2 Antecedent diarrhea history

Ten patients with diarrhea at baseline had documentation of pre‐baseline visits in which diarrhea was assessed. The number of pre‐baseline visits available for these patients ranged from 1 to 7. All of these visits documented the presence of diarrhea. The period between the earliest documented visit with diarrhea and the baseline visit was a median of 4 years with a range of less than 1 year to 30 years.

### 3.3 Response to CDCA therapy

All patients were started on CDCA treatment at the baseline visit. No patient without diarrhea at baseline reported diarrhea during the post‐treatment period. Twenty‐four of the 25 patients with diarrhea at baseline had the absence of diarrhea documented at their first post‐baseline clinic visit (Table 2). The response rate with CDCA for diarrhea resolution was 100% based on at least one post‐baseline visit without diarrhea and 95% as assessed at the first post‐baseline visit. In 68% of cases resolution was complete and sustained as no episodes of diarrhea were documented for follow‐up periods as long as 25 years. Due to the referral nature of the clinic the time between the baseline and first subsequent visit varied substantially (median 14 months, range 2‐151 months) and precluded assessing the timing of symptom resolution.

**TABLE 2:** Response to chenodeoxycholic acid in patients with diarrhea at baseline. Treatment with chenodeoxycholic acid was initiated in all patients at the baseline visit

Patient number	Age at baseline (years)	Timing of first post‐baseline visit (months post‐baseline)	Diarrhea at first post‐baseline visit (Yes/No)	Timing of last post‐baseline visit (months post‐baseline)	Diarrhea at last post‐baseline visit (Yes/No)
1	12	2	No	84	Yes
2	14	5	No	92	No
3	10	6	No	94	No
4	35	7	No	51	No
5	36	151	No	254	No
6	26	5	No	220	No
7	38	10	No	230	No
8	35	138	No	185	No
9	11	33	No	33	No
10	13	9	No	26	No
11	4	4	Yes	109	No
12	8	6	No	110	No
13	13	12	No	164	No
14	2	15	No	210	No
15	46	17	No	89	No
16	8	4	No	67	No
17	31	18	No	180	No
18	32	19	No	180	No
19	46	16	No	16	No
20	32	8	No	158	No
21	37	56	No	156	No
22	17	14	No	80	No
23	20	89	No	131	No
24	40	50	No	297	No
25	33	132	No	304	No

Patients were followed for a median of 9 years (range: 1‐25 years; Table 2) with a median of three visits per patient over the post‐baseline period. Eight patients had visits at which the presence of diarrhea was documented post‐baseline; 7 patients had one such visit, and 1 patient had two positive visits (Table 3). In patients 1 and 10, irritable bowel syndrome was diagnosed as an alternative cause of diarrhea. Patient 11 had a delayed response (>4 months after starting CDCA therapy) for unknown reasons. In patient 18, who was wheelchair bound at baseline, severe constipation resulting in paradoxical diarrhea was diagnosed as the cause of the observed episode of post‐baseline diarrhea. In the remaining patients no clinical reasons for their episode of diarrhea were identified. In seven of the eight cases, one or more visits were negative for diarrhea subsequent to the positive visit. Plasma cholestanol concentrations were documented near or below the upper limit of normal in five of the eight cases at the visit with documented diarrhea, while for three visits cholestenol concentrations were not assessed.

**TABLE 3:** Patients with post‐baseline diarrhea‐positive visits

Patient^a^	Plasma cholestanol at baseline (μmol/L)^b^	Post‐baseline visits)	Diarrhea at visit (Yes/No)	Plasma cholestanol at post‐baseline visit (μmol/L)
1	68	2 months	No	37
		3 months	No	28
		72 months	Yes	7
		84 months	Yes	N/A
6	54	5 months	No	21
		16 months	No	8
		43 months	Yes	14
		84 months	No	N/A
		98 months	No	N/A
		132 months	No	7
		156 months	No	10
		220 months	No	N/A
10	N/A	9 months	No	5
		12 months	Yes	N/A
		26 months	No	N/A
11	28	4 months	Yes	3
		24 months	No	11
		109 months	No	6
17	186	18 months	No	19
		24 months	Yes	N/A
		65 months	No	N/A
		91 months	No	N/A
		93 months	No	N/A
		118 months	No	7
		130 months	No	N/A
		180 months	No	12
18	46	19 months	No	N/A
		65 months	Yes	9
		118 months	No	6
		143 months	No	7
		167 months	No	N/A
		180 months	No	7
20	154	8 months	No	23
		56 months	No	4
		97 months	Yes	N/A
		158 months	No	N/A
22	53	14 months	No	N/A
		23 months	Yes	10
		52 months	No	10
		80 months	No	N/A

*Note:* Individual patients are shown with each post‐baseline visit categorized as diarrhea‐positive or ‐negative. The elapsed time in months from baseline for each visit is shown, with each entry representing an individual visit.

^a^ Patient number corresponds with patients as listed in Table 2.

^b^ Upper limit of normal for plasma cholestanol is 12.5 μmol/L.

Abbreviation: N/A, not available.

One patient, an infant without diarrhea at presentation, had presumed CDCA‐induced hepatotoxicity.^14^ The patient was managed with temporary discontinuation of CDCA therapy, with subsequent reintroduction of CDCA at a dose of 5 mg/kg/day. No other CDCA‐attributable adverse events were reported.

## 4 DISCUSSION

The current retrospective chart review confirms that diarrhea is a common symptom at the time of CTX diagnosis. Initiation of CDCA treatment resulted in at least one diarrhea‐negative visit in 100% of cases. Intractable diarrhea in CTX patients has been recognized since 1991. Cruysberg et al^9^ described three patients who suffered from intractable chronic diarrhea since infancy. In the young age group, neurological symptoms and tendon xanthomas can be absent, so it is important to recognize that diarrhea can be the only symptom in CTX. An early diagnosis of CTX results in an early start of the CDCA therapy, that can prevent the neurological phenotype.^2^, ^3^

The mechanism of CTX‐associated diarrhea is unknown. Deficiency of bile acids can cause steatorrhea, however, van Heijst et al^8^ found no evidence for fat malabsorption, nor a pure secretory‐type of diarrhea. Koopman et al^15^ showed that serum levels of essential fatty acids and fat‐soluble vitamins are normal in CTX patients, indicative of normal fat absorption. It has been hypothesized that the pathological presence of bile alcohols in the lumen of the gut or the intra‐luminal deficiency of CDCA are the most likely causes of diarrhea, perhaps by influencing intestinal motility resulting in an increased transit.^8^

Consistent with the above, several patients in the current cohort had the presence of diarrhea documented for extended periods pre‐CDCA treatment. These included cases with multiple visits over time stretching to as long as 30 years before treatment. These cases illustrate the challenges of establishing a diagnosis of CTX in the absence of a high index of suspicion. Notably there was no evidence of spontaneous remission pre‐treatment, as no visits negative for diarrhea were documented in those patients with visits positive for diarrhea.

It is unclear why some patients with CTX experience diarrhea and others do not. Mean plasma cholestanol concentrations at baseline, the standard biomarker of disordered bile acid metabolism in patients with CTX, were numerically higher in patients with diarrhea as compared to those without. However, there was considerable heterogeneity and overlap in the two cohorts, and the median value was actually lower in those with diarrhea (Figure 1). Patients with diarrhea tended to be younger than those without diarrhea at diagnosis. This may be the result of diarrhea motivating a more comprehensive diagnostic evaluation leading to an earlier diagnosis.

The response rate for diarrhea resolution with CDCA was 100% based on having at least one post‐baseline visit without diarrhea and 95% as assessed at the first post‐baseline visit. In 68% of cases resolution was complete and sustained as no episodes of diarrhea were documented for follow‐up periods as long as 25 years. If patients with a single post‐baseline visit with diarrhea but with one or more subsequent visits without diarrhea are considered as responders, the response rate to CDCA was 95%. The etiology of the rare episodes of diarrhea while on CDCA treatment could not be determined in several patients. As CDCA would not modify non‐CTX causes of diarrhea occurring in a general population, some episodes of non‐CTX induced diarrhea would be expected in long term follow up of patients. The single patient (patient 1, Table 3) with two post‐baseline visits with diarrhea had initial resolution of diarrhea based on two post‐baseline visits during the first year of treatment. Irritable bowel syndrome was diagnosed clinically as an alternative cause of diarrhea in this case.

The current study has limitations due to its retrospective, medical record‐based design centered in a specialty referral clinic. As a result, some visit records lacked sufficient detail or assessments relevant to the current analyses. For example, data on stool frequency and BSS assessments were often not available. The status of the subjects between visits is unknown, and the time between visits was highly variable. Nonetheless, this is the largest dataset focused on CTX‐associated diarrhea currently available.

In conclusion, diarrhea is a common feature of CTX at the time of diagnosis and has often persisted for years without spontaneous remission. Plasma cholestanol concentrations do not appear to be a surrogate marker for the presence of diarrhea at the time of diagnosis. CDCA appears to be a safe and effective treatment for the sustained, symptomatic relief of the diarrhea in patients with CTX.

## CONFLICT OF INTEREST

Aad Verrips received honoraria from serving as a consultant for Leadiant Biosciences.

Bianca M.L. Stelten reports no conflict of interest.

Eric P. Brass is a consultant for Leadiant Biosciences.

## AUTHOR CONTRIBUTIONS

Aad Verrips: concept and design, interpretation of data, drafting the manuscript.

Bianca M.L. Stelten: interpretation of data, critical revision of the manuscript.

Eric P. Brass: concept and design, interpretation of data, drafting of the manuscript.

## INFORMED CONSENT

The study was approved by the The Local Ethics Committee of the Canisius Wilhelmina Hospital Nijmegen, the Netherlands. Informed consent was obtained in all subjects.

## References

[1] Cali JJ , Hsieh CL , Francke U , Russell DW . Mutations in the bile acid biosynthetic enzyme sterol 27‐hydroxylase underlie cerebrotendinous xanthomatosis. J Biol Chem. 1991;266(12):7779‐7783. 2019602 PMC4449724

[2] Stelten BML , Huidekoper HH , van de Warrenburg BPC , et al. Long‐term treatment effect in cerebrotendinous xanthomatosis depends on age at treatment start. Neurology. 2019;92(2):e83‐e95. 30530799 10.1212/WNL.0000000000006731

[3] Yahalom G , Tsabari R , Molshatzki N , Ephraty L , Cohen H , Hassin‐Baer S . Neurological outcome in cerebrotendinous xanthomatosis treated with chenodeoxycholic acid: early versus late diagnosis. Clin Neuropharmacol. 2013;36(3):78‐83. 23673909 10.1097/WNF.0b013e318288076a

[4] Berginer VM , Gross B , Morad K , et al. Chronic diarrhea and juvenile cataracts: think cerebrotendinous xanthomatosis and treat. Pediatrics. 2009;123(1):143‐147. 19117873 10.1542/peds.2008-0192

[5] Duell PB , Salen G , Eichler FS , et al. Diagnosis, treatment, and clinical outcomes in 43 cases with cerebrotendinous xanthomatosis. J Clin Lipidol. 2018;12(5):1169‐1178. 30017468 10.1016/j.jacl.2018.06.008

[6] Wong JC , Walsh K , Hayden D , Eichler FS . Natural history of neurological abnormalities in cerebrotendinous xanthomatosis. J Inherit Metab Dis. 2018;41(4):647‐656. 29484516 10.1007/s10545-018-0152-9

[7] Verrips A , van Engelen BG , Wevers RA , et al. Presence of diarrhea and absence of tendon xanthomas in patients with cerebrotendinous xanthomatosis. Arch Neurol. 2000;57(4):520‐524. 10768627 10.1001/archneur.57.4.520

[8] van Heijst AF , Wevers RA , Tangerman A , Cruysberg JR , Renier WO , Tolboom JJ . Chronic diarrhoea as a dominating symptom in two children with cerebrotendinous xanthomatosis. Acta Paediatr. 1996;85(8):932‐926. 8863874 10.1111/j.1651-2227.1996.tb14189.x

[9] Cruysberg JR , Wevers RA , Tolboom JJ . Juvenile cataract associated with chronic diarrhea in pediatric cerebrotendinous xanthomatosis. Am J Ophthalmol. 1991;112(5):606‐607. 1951610 10.1016/s0002-9394(14)76874-6

[10] Wevers RA , Cruysberg JR , Van Heijst AF , et al. Paediatric cerebrotendinous xanthomatosis. J Inherit Metab Dis. 1992;15(3):374‐376. 1405473 10.1007/BF02435980

[11] Bindl L , Lütjohann D , Lentze MJ , von Bergmann K . Cerebrotendinous xanthomatosis presenting as "chologenic diarrhoea". Acta Paediatr. 2001;90(7):828‐829. 11519995

[12] Verrips A , Dotti MT , Mignarri A , Stelten BML , Verma S , Federico A . The safety and effectiveness of chenodeoxycholic acid treatment in patients with cerebrotendinous xanthomatosis: two retrospective cohort studies. Neurol Sci. 2020;41(4):943‐949. 31863326 10.1007/s10072-019-04169-8 PMC7160076

[13] O'Donnell LJ , Virjee J , Heaton KW . Detection of pseudodiarrhoea by simple clinical assessment of intestinal transit rate. BMJ. 1990;300(6722):439‐440. 2107897 10.1136/bmj.300.6722.439 PMC1662249

[14] Huidekoper HH , Vaz FM , Verrips A , Bosch AM . Hepatotoxicity due to chenodeoxycholic acid supplementation in an infant with cerebrotendinous xanthomatosis: implications for treatment. Eur J Pediatr. 2016;175(1):143‐146. 26156051 10.1007/s00431-015-2584-7 PMC4709371

[15] Koopman BJ , Wolthers BG , van der Molen JC , van der Slik W , Waterreus RJ , van Spreeken A . Cerebrotendinous xanthomatosis: a review of biochemical findings of the patient population in The Netherlands. J Inherit Metab Dis. 1988;11(1):56‐75. 3128689 10.1007/BF01800057

